# Combined endoscopic and laparoscopic anastomosis with lumen-apposing metal stent for treatment of obstructed gastrojejunal anastomotic malignancy

**DOI:** 10.1055/a-2335-6901

**Published:** 2024-06-25

**Authors:** Chaiti Gandhi, Radhika Chavan, Maitrey Patel, Rushil Solanki, Milind Prajapati, Dushyant Bhatt, Sanjay Rajput

**Affiliations:** 1 609456 Ansh Clinic, Ahmedabad, India; 2 Akshar Surgical Hospital, Ahmedabad, India

**Fig. 1:**
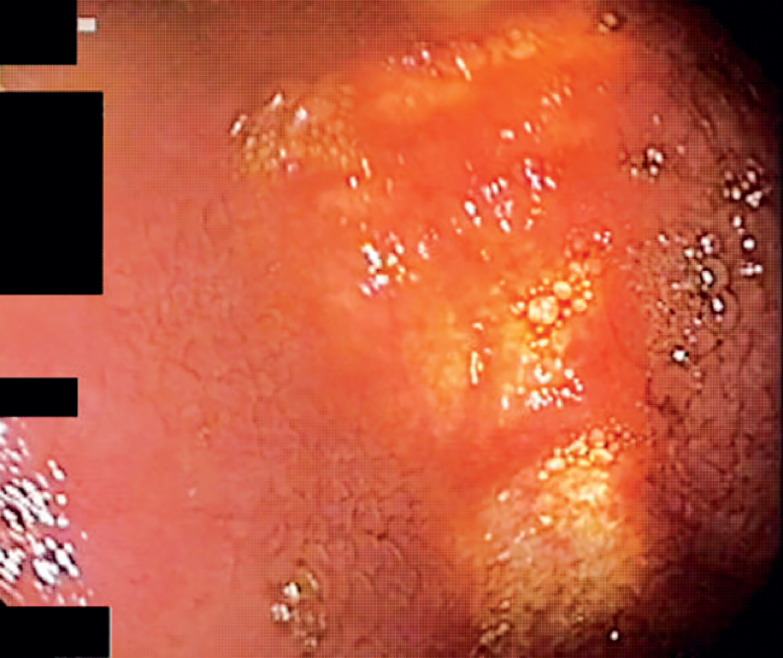
Illumination of the gastric wall by the laparoscope is visualized with the gastroscope.

**Fig. 2:**
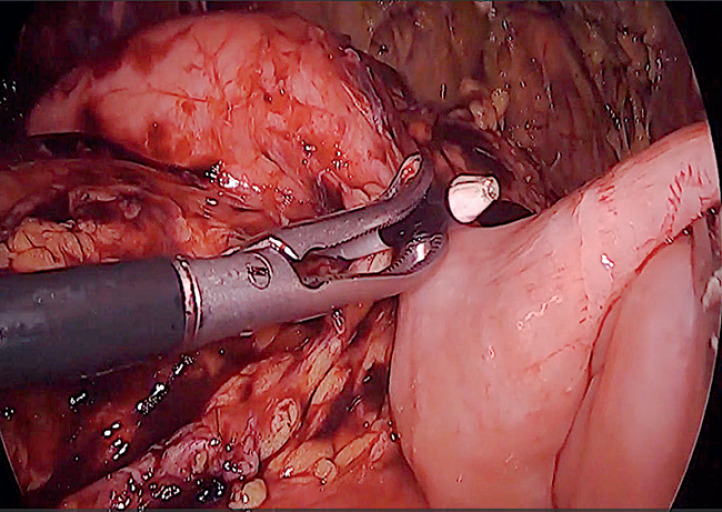
After gastric wall puncture with electrocautery-enhanced lumen-apposing metal stent (LAMS), the delivery catheter is visualized with the laparoscope.

**Fig. 3:**
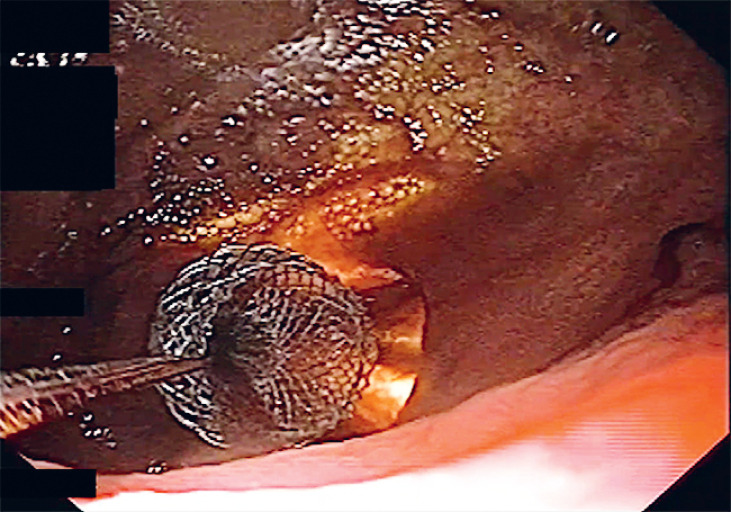
The proximal flange of LAMS is deployed: endoscopic view.

**Fig. 4:**
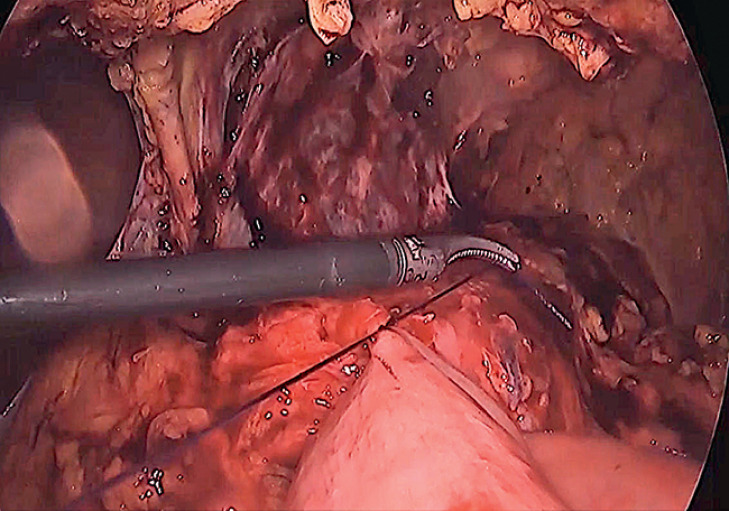
Placement of a suture for close approximation of the stomach and jejunum adjacent to the LAMS.

**Fig. 5:**
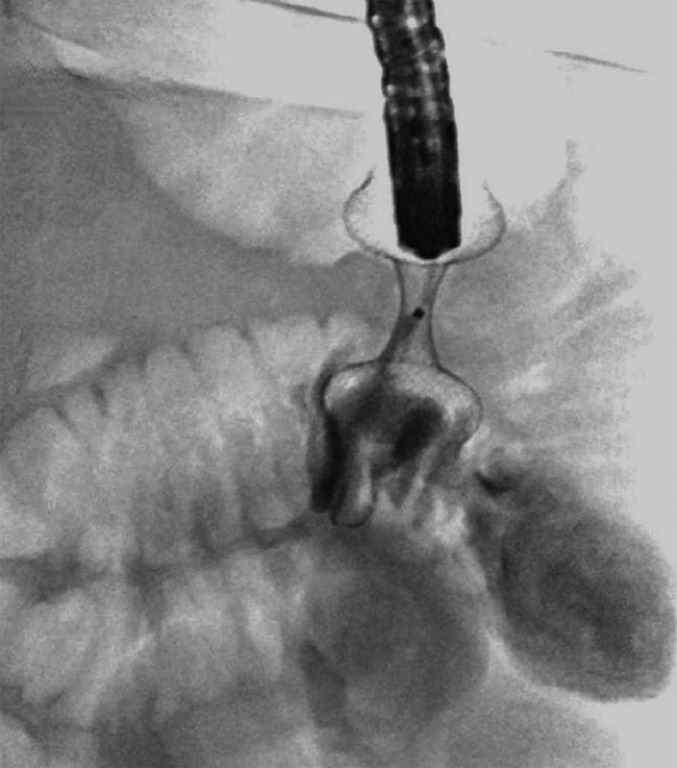
Contrast medium is injected through the LAMS for fluoroscopic confirmation of the LAMS position.

A 75-year-old man with diabetes mellitus, hypertension, ischemic heart disease, and a past history of gastrojejunostomy (40 years back) presented with vomiting and weight loss of 25 kg in 6 months. Gastroscopy showed a proliferative lesion at the anastomotic site with obstructive angulation blocking entry into the efferent loop. Even a guidewire for nasojejunal feeding could not be passed beyond the angulation. Biopsy confirmed moderately differentiated adenocarcinoma. Laboratory evaluation showed moderate anemia (Hb 8.5 g/dL) and hypoproteinemia (total protein 4.6 g/dL, albumin 2.3 g/dL), likely from nutritional deficiency. In view of the patient’s poor nutritional status, our inability to place a catheter for EUS-guided gastrojejunostomy, and the high risk of surgery together with the anticipated duration of recovery, a multidisciplinary team opted for combined endoscopy and laparoscopy-guided gastrojejunostomy with a lumen-apposing metal stent (LAMS). During laparoscopy, the jejunal loop was closely approximated to the stomach with a single suture. Simultaneously, a therapeutic endoscope (GIF-ITQ160; Olympus, Tokyo, Japan) was advanced into the stomach. Gastric wall illumination from the laparoscope was visualized with the gastroscope and gastric wall puncture was guided to a point near the suture site ( Fig. 1 ). The gastric wall was punctured with an electrocautery-enhanced LAMS (20 × 10 mm Hot Axios; Boston Scientific Corp., Massachusetts, USA). Following puncture of the gastric wall, the delivery system was guided with the laparoscope and passed into the jejunal lumen with the cautery tip ( Video 1 , Fig. 2 ). Following deployment of the distal flange, the proximal flange was deployed (after visualization of the black marker) under direct endoscopic view ( Fig. 3 ). With deployment of the LAMS, distension of the collapsed jejunum was immediately noted on laparoscopy. For additional safety, another suture was placed between the gastric and the jejunal wall next to the LAMS ( Fig. 4 ). Accurate placement of the LAMS was also confirmed fluoroscopically by injection of contrast through the LAMS ( Fig. 5 ). The patient was commenced on a liquid diet the following day and was discharged within 24 hours.

Treatment of obstructed gastrojejunal anastomosis with lumen-apposing metal stent under combined endoscopic and laparoscopic guidance.Video 1

Endoscopy- and laparoscopy-guided gastrojejunal anastomosis with a LAMS is safe and feasible and can be considered in patients with altered anatomy and poor nutritional status.

Endoscopy_UCTN_Code_CPL_1AM_2AI

